# Platinum-trimer decorated cobalt-palladium core-shell nanocatalyst with promising performance for oxygen reduction reaction

**DOI:** 10.1038/s41467-019-08323-w

**Published:** 2019-01-25

**Authors:** Sheng Dai, Jyh-Pin Chou, Kuan-Wen Wang, Yang-Yang Hsu, Alice Hu, Xiaoqing Pan, Tsan-Yao Chen

**Affiliations:** 1 Department of Materials Science and Engineering, University of California, Irvine, CA 92697 USA; 2 Department of Mechanical and Biomedical Engineering, City University of Hong Kong, Kowloon, Hong Kong; 3 Institute of Materials Science and Engineering, National Central University, Taoyuan, 32001 Taiwan; 4 Department of Engineering and System Science, National Tsing Hua University, Hsinchu, 30013 Taiwan; 5 Department of Physics and Astronomy, University of California, Irvine, CA 92697 USA; 6 Irvine Materials Research Institute (IMRI), University of California, Irvine, CA 92697 USA; 7 Institute of Nuclear Engineering and Science, National Tsing Hua University, Hsinchu, 30013 Taiwan; 8 Higher Education Sprout Project, Competitive Research Team, National Tsing Hua University, Hsinchu, 30013 Taiwan; 9 Hierarchical Green-Energy Materials Research Center, National Cheng Kung University, Tainan, 70101 Taiwan

## Abstract

Advanced electrocatalysts with low platinum content, high activity and durability for the oxygen reduction reaction can benefit the widespread commercial use of fuel cell technology. Here, we report a platinum-trimer decorated cobalt-palladium core-shell nanocatalyst with a low platinum loading of only 2.4 wt% for the use in alkaline fuel cell cathodes. This ternary catalyst shows a mass activity that is enhanced by a factor of 30.6 relative to a commercial platinum catalyst, which is attributed to the unique charge localization induced by platinum-trimer decoration. The high stability of the decorated trimers endows the catalyst with an outstanding durability, maintaining decent electrocatalytic activity with no degradation for more than 322,000 potential cycles in alkaline electrolyte. These findings are expected to be useful for surface engineering and design of advanced fuel cell catalysts with atomic-scale platinum decoration.

## Abstract (web summary)

Fuel cells are promising for converting fuel into electricity, but rely on development of high-performance catalysts for oxygen reduction. Here the authors report a highly durable platinum-trimer decorated cobalt-palladium catalyst with low platinum loading for electrocatalysis of oxygen reduction.

## Introduction

Fuel cells, which convert chemical energy from a fuel into electricity through an electrochemical reaction, are promising for stationary power generation and transportation, and thus may help solve global problems of energy supply and clean environment^1–4^. However, the sluggish kinetics of the oxygen reduction reaction (ORR) at the cathode, and the high cost of Pt used in electrocatalysts pose obstacles to the commercial viability of this technology^5–7^. In the past decades, substantial efforts have been devoted to developing high-performance electrocatalysts with minimal Pt content, through the control of particle size, morphology, chemical composition, and surface configuration^8–12^. For example, alloying Pt and non-noble metals (e.g., Fe, Co, Ni) reduces the Pt usage in electrocatalysts while improving their intrinsic ORR activity, in comparison with the pure Pt catalysts^12–14^; decorating Pt overlayers on metal nanoparticles is another strategy to achieve decent ORR performance at a low Pt content^15–21^.

Besides the improved ORR activity in these advanced studies^12–21^, there is still room for higher Pt utilization efficiency at the atomic level in ORR catalysts^22^. In addition, it is notable that durability is an even more critical issue of the application of ORR catalysts since the agglomeration and surface corrosion of nanoparticles always result in a rapid loss of the ORR performance^23–25^. Therefore, designing and synthesizing novel ORR catalysts that possess high catalytic activity and durability with a low Pt content remains an important challenge.

In an attempt to address these critical issues of ORR nanocatalysts, we report a new design configuration of a ternary Co-Pd-Pt catalyst at a low Pt loading of 2.4%, where Pt trimer (Pt_3_) species are decorated on a Co-Pd core-shell (Co@Pd) structure. This ternary catalyst shows an ORR mass activity that is enhanced by a factor of 30.6 relative to a commercial Pt catalyst. In particular, the Co-Pd-Pt catalyst exhibits an outstanding durability, maintaining its high activity for over 322,000 potential cycles in alkaline electrolyte due to the survival and dispersion of the decorated Pt_3_ species during ORR. Theoretical calculations reveal a unique charge localization on the ternary catalyst surface, induced by the Pt_3_ decoration, thus weakening the chemisorbed anions and improving the ORR performance. These findings should help pave the way for atomic-scale surface engineering and design of advanced ORR nanocatalysts used in alkaline fuel cells.

## Results

### Sample preparation

A carbon nanotube (CNT)-supported ternary Co-Pd-Pt nanocatalyst (Co@Pd-Pt/CNT), consisting of a Co@Pd core-shell structure and surface decoration of Pt_3_, was synthesized via a developed wet chemical reduction method^26^, following a controlled sequence of Co nanoparticle formation, Pd shell growth, and Pt decoration. Particularly, the reaction time of the final step of Pt decoration was restricted within 10 s for a precise control of the Pt_3_ decoration (detailed synthetic approaches are described in Methods and Supplementary Notes 1 and 2).

In addition, control samples, including CNT-supported Co (Co/CNT), CNT-supported Pt (Pt/CNT), CNT-supported Pd (Pd/CNT), CNT-supported Co-Pd core-shell (Co@Pd/CNT), CNT-supported Co-Pt core-shell (Co@Pt/CNT), and CNT-supported Pd-Pt core-shell (Pd@Pt/CNT) catalysts were also prepared through similar synthetic procedures for comparison and analysis.

### Electron microscopy

**Fig. 1:**
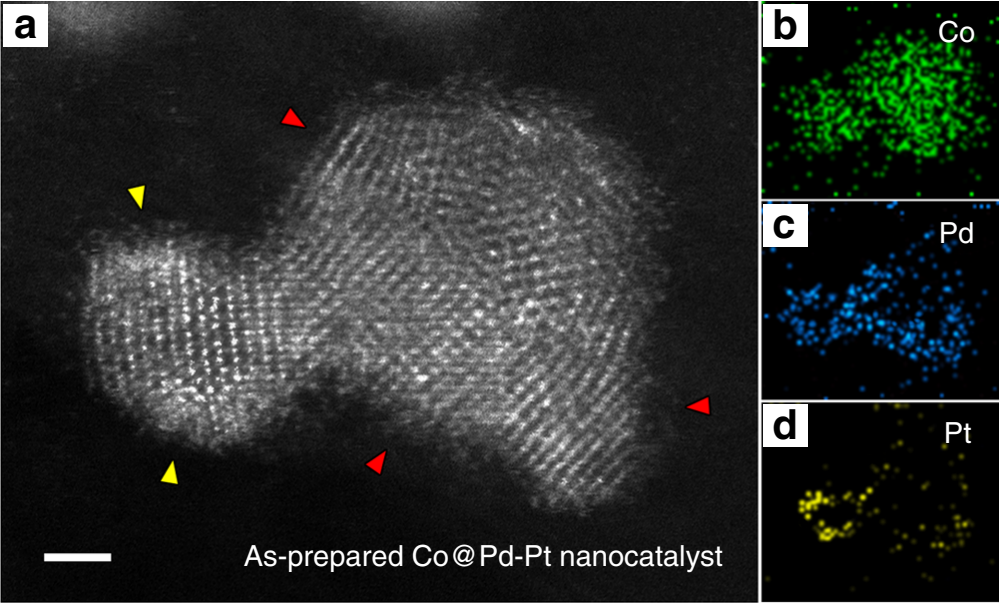
Scanning transmission electron microscopy (STEM) of Pt-decorated Co-Pd. **a** Atomic-scale high angle annular dark field-STEM (HAADF-STEM) image of as-prepared Pt-decorated Co-Pd (Co@Pd-Pt) nanocatalyst. Yellow and red arrows indicate the decorated Pt species and the unconformable Pd shell regions, respectively. Scale bar: 1 nm. **b**–**d** EDS elemental maps of the Co@Pd-Pt nanocatalyst in **a**

Aberration-corrected scanning transmission electron microscopy (AC-STEM) was utilized to reveal morphology and structure information of the as-synthesized Co@Pd-Pt/CNT nanocatalyst. Figure 1 shows the typical result of high angle annular dark field (HAADF)-STEM imaging and energy dispersive X-ray spectroscopy (EDS). As illustrated by the EDS elemental maps in Fig. 1b, c, a structure of a Co-rich core and a Pd-rich shell is well reflected (more STEM results showing the Co@Pd core-shell structure are provided in Supplementary Fig. 1). According to the atomic-scale STEM image, the Pd shell exhibits a unconformable characteristic, showing various crystal orientations on the Co core, as indicated by the red arrows in Fig. 1a. Moreover, the decorated Pt species can be directly observed in the HAADF-STEM image due to the highest Z-contrast^27^ (indicated by the yellow arrows in Fig. 1a), and further confirmed by the EDS elemental map in Fig. 1d. Based on typical STEM results (see Supplementary Figs. 1 and 2), it is noticeable that the decorated Pt species show a fuzzy feature, instead of clear lattice fringes, indicating its unique structure on the Co@Pd nanoparticle surface.

According to the semi-quantitative EDS analysis, chemical composition of the Co@Pd-Pt nanoparticles is revealed as 52.1% of Co, 43.4% of Pd, and 4.5% of Pt (weight ratio). Moreover, X-ray photoelectron spectroscopy (XPS) shows that the surface composition of the as-synthesized nanoparticles is 3.9% of Co, 49.2% of Pd, and 46.9% of Pt on average, within a probing depth around 1.5 nm (see Supplementary Figs. 3–5 and Supplementary Table 1), confirming the structure of surface Pt-decorated Co-Pd core-shell nanoparticles.

### X-ray absorption spectroscopy

**Fig. 2:**
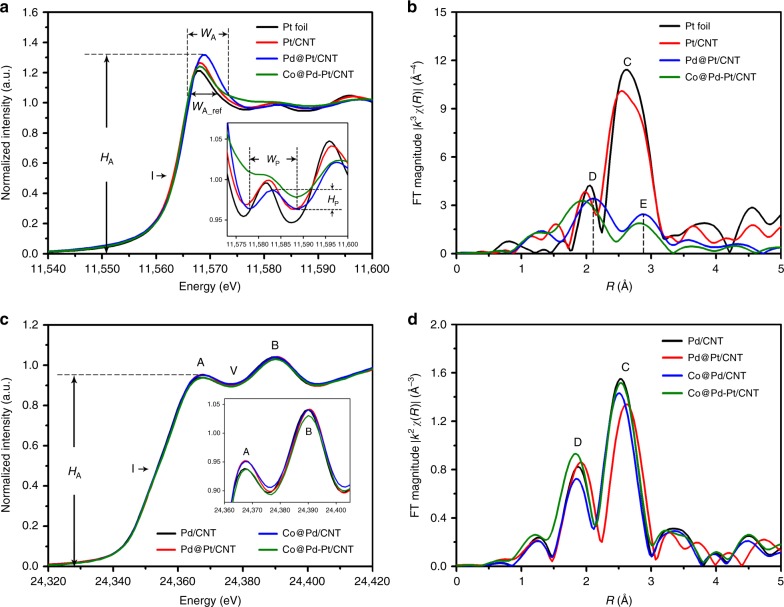
X-ray absorption spectra of Pt-decorated Co-Pd and control samples. **a** X-ray absorption near-edge spectra (XANES) of Pt L_3_-edge of standard Pt foil, Pt/carbon nanotube (CNT), Pd-Pt core-shell/CNT (Pd@Pt/CNT), and Pt-decorated Co-Pd/CNT (Co@Pd-Pt/CNT) catalysts. The inset shows the enlarged post-edge region. **b** Fourier-transformed extended X-ray absorption fine structure (FT-EXAFS) of the four samples in **a**. **c** XANES spectra of Pd K-edge of Pd/CNT, Pd@Pt/CNT, Co-Pd core-shell/CNT (Co@Pd/CNT), and Co@Pd-Pt/CNT catalysts. The inset shows the enlarged region of peaks A and B. **d** FT-EXAFS of the four samples in **c**

For a deeper understanding of the unique structure of the ternary Co@Pd-Pt catalyst, X-ray absorption spectroscopy (XAS) was carried out while representative control samples were also analyzed. For example, Fig. 2a shows the normalized Pt L_3_-edge X-ray absorption near-edge spectra (XANES) of Co@Pd-Pt/CNT catalyst, while the spectra of Pt/CNT, Pd@Pt/CNT, and the reference sample (Pt foil) are also provided for comparison. As indicated by the black arrow in Fig. 2a, the position of the inflection point in XANES refers to the threshold energy for 2*p* to 5*d* electron transition, and is generally proportional to the oxidation state of Pt. Clearly, positions of the inflection point of Co@Pd-Pt/CNT, Pd@Pt/CNT, and Pt/CNT catalysts are all identical to that of Pt foil, demonstrating the metallic characteristics of Pt atoms in these materials (more details can be found in Supplementary Fig. 6 showing the derivative XANES spectra in the near-edge region). Moreover, the relative extent of empty states and the splitting of Pt 5*d*_5/2_ orbital can be revealed through the intensity (*H*_A_) and the width (*W*_A_) of the main absorption peak, as illustrated in Fig. 2a. For example, compared to that of Pt foil, the absorption intensity of Pt/CNT sample (the red line) show an increment, corresponding to the oxygen chemisorption due to an absence of surface stabilization. As for the Pd@Pt/CNT sample (the blue line), a substantial increment of the absorption intensity shows that majority of the coordination sites of Pt atoms are occupied by oxygen chemisorption in this catalyst sample. However, the Co@Pd-Pt/CNT nanocatalyst (the green line) represents a much lower *H*_A_, demonstrating a strong charge localization from the Co@Pd surface to the decorated Pt species, while the narrowest width *W*_A_ reflects the lowest extent of 5*d*_5/2_ splitting among these catalyst samples.

In addition, features in the post edge of XANES spectra, corresponding to the interference of photoelectrons with local atoms, are usually used to describe the extent of local structural ordering around the target atom. As shown in the inset of Fig. 2a, a major difference is revealed between the Pt-decorated catalysts (Pd@Pt/CNT and Co@Pd-Pt/CNT) and the Pt-based samples (Pt/CNT and Pt foil) that the intensity of the oscillation hump (*H*_p_) decreases along with broadening of the width (*W*_p_) of the hump in the previous two catalysts, indicating disordered structures around the decorated Pt species. Particularly, a significantly attenuated *H*_p_ and a broadened *W*_p_ of the Co@Pd-Pt/CNT catalysts (see the green line in the inset of Fig. 2a) denote the presence of high-density local defects around Pt atoms on the Co@Pd surface.

Quantitative atomic structural parameters around the Pt atoms are further revealed by model analysis of extended X-ray absorption fine structure (EXAFS). Figure 2b presents the Fourier-transformed extended X-ray absorption fine structure (FT-EXAFS) spectra (radial structure function, RSF) of Pt L_3_-edge of the four samples, and corresponding structural parameters are summarized in Table 1 (more details including the comparison between fitting curves and experimental spectra are provided in Supplementary Fig. 7). For example, in the RSF of Pt/CNT, the radial peak (C) at 2.75 Å accounts for the X-ray interference with metallic Pt-Pt bond of a coordination number (CN) of 6.45. In the RSF of Pd@Pt/CNT sample, it is clear that the main radial peak (1.9–3.1 Å) is split into peaks D and E, as illustrated in Fig. 2b, featuring an interference between photoelectrons and both homo-atomic Pt-Pt (at a distance (*R*_Pt-Pt_) of 2.705 Å and a CN (CN_Pt-Pt_) of 4.49) and hetero-atomic Pt-Pd (*R*_Pt-Pd_ = 2.662 Å and CN_Pt-Pd_ = 1.46) bond pairs in the Pt-decorated Pd nanoparticles^28^. The radial peak with a bond length of 1.893 Å represented the oxygen chemisorption bond in Pt atoms. According to a low CN_Pt-O_ (0.57) and a substantially smaller total CN of Pt atoms (CN_total_Pt_ = 5.95) than that of surface atoms in an ideal crystal (CN_total_surface_ = 6–9 at different facets), it can be revealed that the Pt clusters were intercalated in the top layer of the Pd crystal in the Pd@Pt/CNT catalysts.

**Table 1:** Quantitative results of X-ray absorption spectroscopy model analysis at Pt L_3_-edge and Pd K-edge of Co@Pd-Pt/CNT nanocatalysts and control samples

Sample	Pt L_3_-edge				Pd K-edge			
	Bond pair	CN	*R*	*χ*	Bond pair	CN	*R*	*χ*
Co@Pd-Pt	Pt-Pt	1.95	2.691	43.5%	Pd-Pd	5.88	2.762	62.3%
	Pt-Pd	1.16	2.671	25.9%	Pd-Pt	2.91	2.711	30.8%
	Pt-Co	1.37	2.629	30.6%	Pd-Co	0.65	2.708	6.9%
Co@Pd					Pd-Pd	5.51	2.731	89.9%
					Pd-Co	0.62	2.753	10.1%
	Pt-O	0.57	1.893		Pd-Pd	5.5	2.75	66.7%
Pd@Pt	Pt-Pt	4.49	2.705	75.5%	Pd-Pt	2.75	2.723	33.3%
	Pt-Pd	1.46	2.662	24.5%	Pd-O	0.47	2.03	
Pt/CNT	Pt-Pt	6.45	2.752					
Pd/CNT					Pd-Pd	5.71	2.747	
					Pd-O	0.78	2.041	

*CNT* carbon nanotube

*χ* represents a hetero-atomic intermix of coordination neighbors to the target atom in a selected bond pair. For the Pt L_3_-edge result, *χ*_M_ = (CN_Pt-M_ / CN_Pt-total_) × 100%, where M represents neighboring atoms of Pt and CN_Pt-total_ = Σ CN_Pt-M_ (for instance, CN_Pt-total_ = CN_Pt-Pt_ + CN_Pt-Pd_ for the sample of Pd@Pt catalyst)

*R*-factor is 0.04 for Co@Pd-Pt, 0.01 for Pd@Pt, 0.01 for Pt/CNT at Pt L_3_- edge; 0.02 for Co@Pd-Pt, 0.01 for Pd@Pt,; and 0.01 for Pd/CNT at Pd K-edge

As for the result of Co@Pd-Pt/CNT nanocatalyst (see the green line), radial peaks across 1.7–3.2 Å were attributed to Pt-Co (*R*_Pt-Co_ = 2.629 Å, CN_Pt-Co_ = 1.37), Pt-Pd (*R*_Pt-Pd_ = 2.671 Å, CN_Pt-Pd_ = 1.16), and Pt-Pt (*R*_Pt-Pt_ = 2.691 Å, CN_Pt-Pt_ = 1.95) bond pairs. Particularly, the CN of Pt-Pt bonds is revealed as 1.95, much smaller than that in the Pd@Pt structure. This evidence demonstrates the formation of Pt_3_ (possessing an average CN of 2.0 theoretically) on the Co@Pd nanoparticle surface in the as-synthesized ternary catalyst. Although the Pt decorations look like nanometer-range clusters in our STEM observation (see Fig. 1a), they are actually the agglomerated Pt_3_ species, and that is the reason why lattice fringes are not visible in the atomic-scale STEM image. Moreover, it is found that the hetero-atomic intermix of Co to Pt atoms (*χ*_Co_ = 30.6%) is higher than that of Pd to Pt atoms (*χ*_Pd_ = 25.9%), indicating a higher extent of galvanic replacement of Pt^4+^ to Co atoms (Pt^4+^ + Co^0^_core_ → Pt^0^_cluster_ + Co^2+^) during the synthesis process. Notably, the Co@Pd-Pt/CNT catalyst shows a higher total hetero-atomic intermix (*χ*_Co_ + *χ*_Pd_ = 56.5%) than that of Pd@Pt/CNT catalysts (*χ*_total_ = *χ*_Pd_ = 24.5%), demonstrating that the Pt_3_ species are sitting at the defective regions between the Co core and the Pd shell of Co@Pd nanoparticles.

Figure 2c presents the Pd K-edge XANES spectra of the Co@Pd-Pt/CNT nanocatalyst and the control samples (Pd/CNT, Co@Pd/CNT, and Pd@Pt/CNT). It is clear that the binary and ternary catalysts are all showing identical positions of inflection point (I), two resolved peaks (A and B), and valley (V) to those of Pd/CNT, demonstrating the metallic state of Pd atoms in these samples^29^. Particularly, as shown in the enlarged inset, the Co@Pd-Pt/CNT catalyst (the green line) represents the lowest intensities of both peaks A and B, reflecting its lowest empty state in Pd 4*s*/4*p* orbital, which is attributed to the steric coverage of Pt_3_ species at the defect sites of Pd shell (near the Co core).

Moreover, Fig. 2d presents the *k*^2^-weighted RSF pattern of Pd K-edge of these four samples, and the fitting curves with experimental EXANES spectra are provided in Supplementary Fig. 8. Similar to the analysis of Pt L_3_-edge, the radial peak C in Fig. 2d is corresponding to an interference between X-ray and metallic Pd-Pd bond pairs, while the peak D is a typical out-of-phase X-ray interference of bond pairs between heavy transition metal atoms. Taking the Pd/CNT as a reference, the Co@Pd structure shows a decreasing intensity of peak C, confirming the unconformable structure of Pd on the Co surface. However, while the Pt_3_ species are decorated on the Co@Pd surface, the intensity of peak C of Co@Pd-Pt was enhanced, close to that of metal Pd (Pd/CNT). This result indicates that Pd shell (on the Co core) tends to form a locally ordered structure with the decoration of Pt_3_, although it exhibits an unconformable characteristic within a long range, as we observed in the STEM images.

Combining the above XAS analysis of both Pt L_3_-edge and Pd K-edge, it is notable that the Pt-Pt bond and Pd-Pd bond of Co@Pd-Pt structure were compressed and elongated at the greatest extent, respectively (see the parameters in Table 1). Such obvious strain effect is likely to introduce charge relocation, and may affect the catalytic performance accordingly.

### Ultraviolet photoelectron spectroscopy

**Fig. 3:**
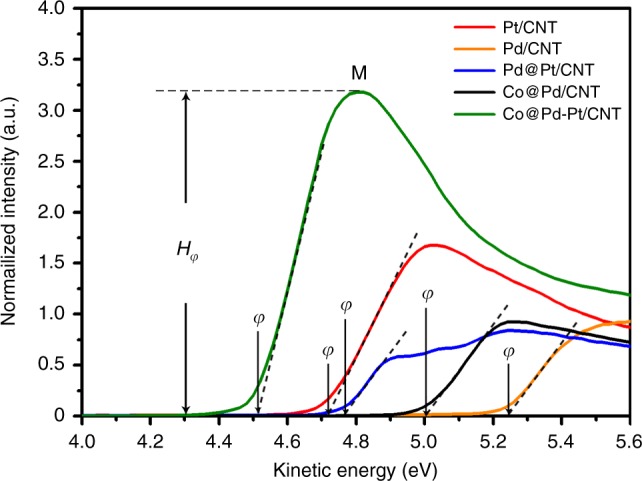
Ultraviolet photoelectron spectra of Pt-decorated Co-Pd and controls. The work function of Pt-decorated Co-Pd (Co@Pd-Pt/carbon nanotube (CNT)) catalyst is lower than that of the control samples including Pt/CNT, Pd/CNT, Pd-Pt core-shell (Pd@Pt/CNT), and Co-Pd core-shell (Co@Pd/CNT) catalysts

Ultraviolet photoelectron spectroscopy (UPS) was carried out to determine the work function (*φ*) to elucidate the charge localization of the Co@Pd-Pt/CNT nanocatalyst. Work function refers to the energy that is required to remove a valance electron to infinity from a material surface of a given solid, and is a qualitative index to describe the activity of materials in a redox reaction. In general, *φ* of metallic nanoparticles is always dominated by two factors: particle size and surface configuration^30,31^. Here, Fig. 3 compares the UPS spectra of various catalyst samples, and *φ* is determined by the position of secondary cutoff potential. Accordingly, the work function follows a trend of Co@Pd-Pt/CNT (4.51 eV) < Pt/CNT (4.74 eV) < Pd@Pt/CNT (4.77 eV) < Co@Pd/CNT (5.01 eV) < Pd/CNT (5.23 eV). Based on our STEM statistic observation (see Supplementary Table 2), it is revealed that the average size of Pd@Pt, Co@Pd, and Co@Pd-Pt nanoparticles are quite similar, within the range of 4.12–4.65 nm. However, the Co@Pd-Pt nanoparticles show a much lower work function than those two core-shell structures, indicating a unique surface effect from the Pt_3_ decoration. Also, a strong charge localization on the Co@Pd-Pt surface was confirmed according to the substantially enhanced intensity (*H*_*φ*_) of the peak M in the near-Fermi level (Fig. 3), highlighting the quantum size effect of decorated Pt_3_ species^32^. This finding is consistent with our XAS result showing a high rate of 5*d*_5/2_ occupancy of the Pt atoms in Co@Pd-Pt/CNT catalyst.

These experimental evidences are further supported by our density functional theory (DFT) calculations. In order to mimic the structure and configuration of Co@Pd-Pt nanoparticles as revealed from our AC-STEM, XAS and additional X-ray diffraction (see Supplementary Fig. 9 and Supplementary Table 3) results, a model of five layers of 4 × 4 Co (111) slab capping by one layer of Pt_3_-doped Pd slab was constructed (more details are provided in the calculation part of Methods). As a result, a strong electron coupling effect was revealed by the presence of a localized charge bridging the Pt and Co atoms, as illustrated in Supplementary Fig. 10, in agreement with the enhanced *H*_*φ*_ of Co@Pd-Pt nanocatalyst in the UPS result. A strong negative field is formed around the Pt_3_ decoration, and may weaken the adsorption of O_2_/O at the Pt_3_ site, thus facilitating the ORR.

### Electrochemical testing

To assess the ORR catalytic performance of Co@Pd-Pt/CNT nanocatalyst, linear sweep voltammetry (LSV) and cyclic voltammetry (CV) were carried out. ORR polarization curves of Co@Pd-Pt/CNT nanocatalysts and various control samples are provided in Supplementary Fig. 11. Electrochemical active surface areas (ECSAs) are calculated based on corresponding CV curves by using the oxygen desorption peak in the backward potential sweeping curve (detailed ECSA data of various ORR catalysts are listed in Supplementary Table 4).

**Fig. 4:**
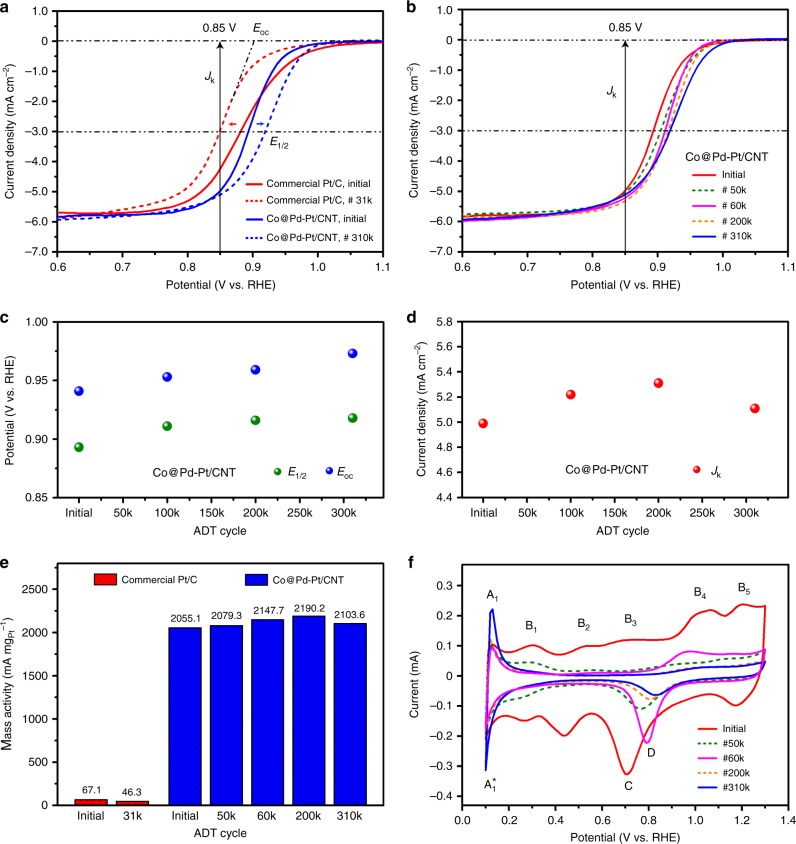
Electrochemical results of Pt-decorated Co-Pd nanocatalyst. **a** Initial and final oxygen reduction reaction (ORR) polarization curves of commercial Pt/C and Pt-decorated Co-Pd (Co@Pd-Pt/carbon nanotube (CNT)) catalysts. **b** ORR polarization curves of Co@Pd-Pt/CNT at selected accelerated degradation test (ADT) cycles. **c** Changes of onset potential and half-wave potential of Co@Pd-Pt/CNT at selective ADT cycles. **d** Changes of kinetic current density of Co@Pd-Pt/CNT at selective ADT cycles. **e** Comparison of ORR mass activity of commercial Pt/C and Co@Pd-Pt/CNT at selected ADT cycles. **f** Cyclic voltammetry (CV) curves of Co@Pd-Pt/CNT at selected ADT cycles

Figure 4a shows a comparison of ORR polarization curves of Co@Pd-Pt/CNT and commercial Pt/C catalyst (J.M. Pt), which were normalized by the area of carbon electrode. At the initial state (see the solid lines), Co@Pd-Pt/CNT catalyst shows an enhanced half-wave potential (*E*_1/2_) to the commercial Pt/C, indicating a smaller energy barrier for the ORR. The onset potential (*E*_oc_) of Co@Pd-Pt/CNT nanocatalyst is 0.944 V vs. a reversible hydrogen electrode (vs. RHE), a little bit lower than that of commercial Pt/C (0.956 V vs. RHE), because of the oxide species on its surface (see the following CV analysis). In order to compare the activity for these two catalysts, current densities at 0.85 V vs. RHE (denoted as *J*_k_) are normalized with respect to the loading amount of metal Pt. As illustrated in Fig. 4e, the Co@Pd-Pt/CNT catalyst exhibits an initial mass activity of 2055.1 mA mg_Pt_^−1^ at 0.85 V vs. RHE, representing an improvement by a factor of 30.6 relative to the commercial Pt/C catalyst (67.1 mAmg_Pt_^−1^ at 0.85 V vs. RHE), while at a low Pt loading of 2.4% (detailed calculation of mass activity can be found in Supplementary Note 3, and more ORR mass activity results of the control samples are provided in Supplementary Table 5).

The long-term durability of Co@Pd-Pt/CNT catalyst was first evaluated by conducting the accelerated degradation test (ADT) up to 310,000 cycles (more detailed ADT conditions can be found in Methods). As shown in the representative ORR curves at selected ADT cycles in Fig. 4b, the ORR performance were largely retained with no degradation, even after 310k ADT cycles. For example, both *E*_oc_ and *E*_1/2_ show a slight increase during the ADT cycling (Fig. 4c), indicating the additional gain of the active sites; *J*_k_ was always within a range of 5.0–5.3 mA cm^−2^, showing no obvious deterioration as compared to the initial state (Fig. 4d).

For a direct comparison, the final state of ORR curves of Co@Pd-Pt/CNT catalyst (#310k cycle) and commercial Pt/C catalyst (#31k cycle, since *J*_k_ was <75% of the initial state) are plotted in Fig. 4a. Clearly, the commercial Pt/C catalyst exhibits a negative shift (−32.2 mV vs. RHE) of the half-wave potential after 31k cycles, denoting a degradation of its ORR performance due to the particle agglomeration^25^. However, in contrast, our Co@Pd-Pt/CNT catalyst shows a positive shift (+27.1 mV vs. RHE) of the half-wave potential after continuous 310k ADT cycles, demonstrating an outstanding durability, and even an enhancement of its ORR performance.

In addition, Fig. 4e summarizes the change of mass activity of Co@Pd-Pt/CNT catalyst during ADT cycling, and the commercial Pt/C is used as a baseline catalyst for comparison. The mass activity of the commercial Pt/C degraded steadily by 30% after 31k cycles, due to the agglomeration of Pt nanoparticles (see our TEM result in Supplementary Fig. 12). However, it is surprising to find that the mass activity of the Co@Pd-Pt/CNT catalyst always maintained at a very high level, as illustrated in Fig. 4e. Notably, the mass activity was still as high as 2103.6 mA mg_Pt_^−1^ at 0.85 V vs. RHE after the #310k ADT cycle, suggesting this is not the limit of the Co@Pd-Pt/CNT catalyst.

## Discussion

The Pt_3_-decorated Co@Pd catalyst shows desirable ORR activity and durability simultaneously, as compared to the advanced Pt-decorated metal catalysts in alkaline media from the literature^33–36^, in which the highest mass activity is at a level of about 400 mA mg_Pt_^−1^ at 0.85 V vs. RHE and the degradation always takes place around 5k cycles (more detailed comparisons are summarized in Supplementary Table 6). Clearly, in contrast to the Pt overlayers and large Pt clusters on metal nanoparticles^33–36^, the atomic-scale Pt decoration^26^ on Co@Pd nanoparticle not only shows a unique surface configuration at a higher Pt utilization efficiency, but also provides a further improved promotion effect toward ORR.

To have a better understanding of the specific advantages from the Pt_3_ decoration, we first analyze the CV curves of the Co@Pd-Pt catalyst that were collected during the ADT cycling, as shown in Fig. 4f (more detailed CV curves at every 10,000 of the first 70,000 cycles are provided in Supplementary Fig. 13). At the initial state (the red solid line), current peaks corresponding to the redox of the metal nanoparticle surface can be revealed: for example, the five current peaks (B_1_–B_5_), as marked in the forward sweeping curve, are attributed to the formation of oxide species with different combinations of Co, Pd, and Pt, while the obvious peak C in the backward sweeping is determined as reduction of PdO, according to the literature^37,38^ as well as our CV results of control samples and theoretical calculation (more details are expanded in Supplementary Note 4 on the basis of Supplementary Fig. 14 and Supplementary Tables 7 and 8). As the ADT going on, an obvious evolution of the oxide reduction peak C is found during the first 50k cycles: the peak intensity was decreasing while the peak position was gradually shifting to the high potential region, reflecting a catalyst surface reconstruction. Meanwhile, the redox peaks at hydrogen underpotential deposition (*H*_upd_) region, as indicated by A_1_ and A_1_^*^ in Fig. 4f, were becoming narrow and sharp at the position of ~0.1 V vs. RHE, showing a unique hydrogen adsorption/desorption characteristic of the Co@Pd-Pt catalyst. According to the published results in literature, this kind of strong H_2_ evolution activity is a typical feature of sub-nanometer Pt clusters^39–41^, consistent with the decorated Pt_3_ species on the Co@Pd surface.

Then, between the interval from #50k to #60k cycles, the oxide reduction peak C continued to shift to the high potential region, but the intensity was greatly enhanced at #60k ADT cycles, as denoted by peak D, which can be determined as reduction of PtO^37,38^. In view of the gradually enhanced peaks A_1_ and A_1_^*^, it can be concluded that the oxygen reduction activity at the catalyst surface was dominated by Pt phase at that time, instead of the initial Pd phase. Afterwards, the shape of CV curves was relatively stable during the subsequent ADT cycles (from #60k to #310k) and the peak intensities of A_1_ and A_1_^*^ were continuously becoming stronger, demonstrating that the sub-nanometer Pt decoration still survived with a further increment of their surface area.

**Fig. 5:**
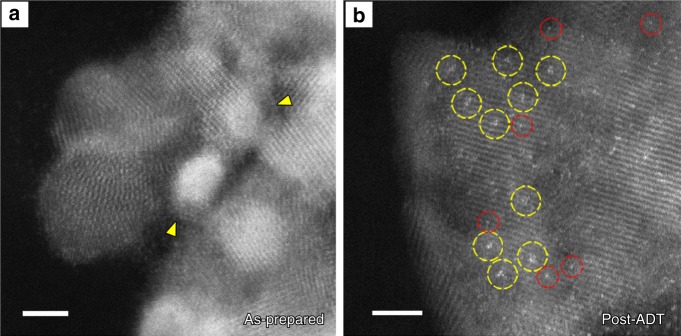
Dispersion of Pt trimer species on the Co-Pd surface. **a** High angle annular dark field-scanning transmission electron microscopy (HAADF-STEM) image of the as-prepared Pt-decorated Co-Pd (Co@Pd-Pt) catalyst. Yellow arrows indicate the agglomerated Pt trimer species. Scale bar: 2 nm. **b** HAADF-STEM image of the Co@Pd-Pt catalyst after 310,000 accelerated degradation test (ADT) cycles. Yellow and red circles indicate the dispersed Pt trimer species and single Pt atoms, respectively. Scale bar: 2 nm

For a direct visualization of the survival of the Pt decoration, atomic-scale STEM characterization was carried out on the post-ADT Co@Pd-Pt/CNT catalyst that was collected after 310,000 ADT cycles. As a comparison, Fig. 5 presents the atomic-scale HAADF-STEM images of the as-prepared Co@Pd-Pt/CNT catalyst (Fig. 5a) and the post-ADT one (Fig. 5b). Obviously, the distribution of Pt_3_ species was changing during the ADT cycling, validating the proposed surface reconstruction: the initially agglomerated Pt_3_ species (as indicated by yellow arrows in Fig. 5a) dispersed into isolated atomic-scale Pt species on the Co@Pd nanoparticle surface. Particularly, the predominant Pt species in the post-ADT sample were revealed as isolated Pt_3_, which can be directly observed in Fig. 5b (as highlighted by yellow circles), while some Pt single atoms (Pt_1_) were also found (as marked by red circles). Quantitative analysis regarding the size of Pt_3_ species are expanded in Supplementary Note 5. Based on STEM data with higher statistics (e.g., see Supplementary Fig. 15), the density of Pt_3_ in the post-ADT catalyst is estimated to be as high as 4.6 Pt_3_/100 nm^2^, indicating that majority of Pt_3_ decoration survived on the Co@Pd nanoparticle surface after over 300,000 ADT cycles.

Actually, the defective region between the Co core and the unconformable Pt shell plays an important role in dispersing the Pt_3_ species. As revealed through the STEM and XAS results, decorated Pt_3_ species prefer to sit at the defective regions between the Co core and the Pt shell. Therefore, since considerable amount of defect sites are generated on the Co@Pd surface during ORR due to decomposition of Pd/Co oxides and desorption of oxygen species, the Pt_3_ species tend to relocate at the new defective sites to minimize the surface energy. This behavior is the origin for enhancement of ORR activity that was observed in the Co@Pd-Pt catalyst during the continuous potential sweeping.

For a further exploration of the stability of the Pt_3_ species on the Co@Pd surface, we stored the rotation electrode (after 310,000 ADT cycles) in distilled water for a couple of days to restore its pH value, and then continued the electrochemical testing for additional 12,000 ADT cycles; therefore, 322k cycles in total. Consequently, the Co@Pd-Pt catalyst was still highly active, representing a stable mass activity (>2000mAmg_Pt_^−1^ at 0.85 V vs. RHE) after #322k ADT cycle, as compared to the state before storage. This result further highlights the outstanding durability of the Co@Pd-Pt/CNT catalyst, which is promising in practical and commercial applications of alkaline fuel cells.

**Fig. 6:**
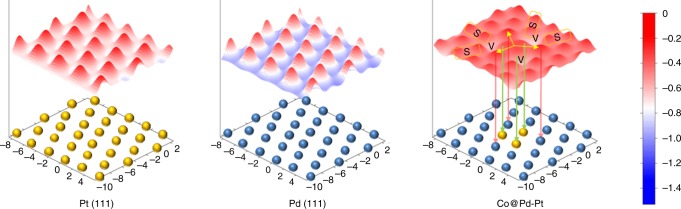
Contours of oxygen adsorption energy. *E*_ads_^O^ in models of Pt (111), Pd (111), and Pt-decorated Co-Pd catalyst surface. Atomic structures of the topmost layers are shown in schematics in which blue and yellow spheres correspond to Pd and Pt atoms, respectively. Co atoms are not shown in the Pt-decorated Co-Pd (Co@Pd-Pt) model

According to our experimental evidence, it is confirmed that the decorated Pt_3_ species are stable and dominate the surface oxygen redox of Co@Pd-Pt/CNT catalyst. Going a step further for deeper mechanistic insights, DFT calculation revealed the ORR promotion effect of the Pt_3_ decoration on the Co@Pd surface (more calculation details are provided in Methods). As shown by the result in Supplementary Fig. 10, both the charge localization around Pt_3_ and the electron-coupling effect between Pt and Co atoms are revealed, thus high density of electrons were extracted in the near-Fermi level. Hence, a strong negative electric field is induced and weakens the adsorption of O/O_2_ at the Pt_3_ and its neighboring sites. Furthermore, we calculated the oxygen adsorption energy (*E*_O_^ads^) on the surface of Co@Pd-Pt catalyst as well as Pt (111) and Pd (111) surfaces, and the result is illustrated in Fig. 6. Accordingly, it is clear that *E*_ads_^O^ peaks and *E*_ads_^O^ valleys are distributed periodically in the contours of Pt (111) and Pd (111) surfaces. However, the presence of slight gradient of *E*_O_^ads^ is found on the Co@Pd-Pt surface, indicating feasible relocation pathways for the O^ads^ between O_2_ splitting sites (site S) and O reduction sites (site V). Meanwhile, relocation of O^ads^ to the neighboring sites reduces the possibility of passivation on Pt_3_ by intermediates (e.g., the suppressed electric double layer current in the CV curves). This characteristic enables the neighboring sites to share the loading from the Pt_3_ in subsequent reaction steps, and maintain the high ORR activity of the Co@Pd-Pt/CNT catalyst.

Furthermore, it is found that the adsorption energy for O_2_ molecule (*E*_ads_^O2^) is very close to 0 on most surface sites of Co@Pd-Pt (see the data in Supplementary Tables 9 and 10). This characteristic implies a substantially weakened O_2_ adsorption on the Co@Pd-Pt surface, which exhibits an oxidation resistance, as compared to the Pt (111) or the Pd (111) surface, thus improving the durability of the Co@Pd-Pt/CNT catalyst. Notably, the theoretical findings that the Pt_3_ decoration not only reduces the Pt loading, but also provides a unique charge localization state that greatly improves the ORR performance.

In conclusion, we designed and synthesized a ternary nanocatalyst with low Pt-loading that consists of a Co@Pd core-shell structure and surface decoration of Pt_3_ species to catalyze ORR in alkaline electrolytes. Advanced characterization result and theoretical calculation revealed a unique charge localization induced by the Pt_3_ decoration at the interfacial region between Co core and Pd shell. Accordingly, the Co@Pd-Pt catalyst shows an ORR mass activity improved by a factor of 30.6 relative to a commercial Pt catalyst. In particular, this ternary catalyst exhibits an outstanding durability, maintaining the high activity, without degradation, for more than 322,000 potential cycles in alkaline electrolytes, due to the decorated Pt_3_ species that becomes dispersed and stabilized on the Co@Pd surface under working condition.

The Co@Pd-Pt catalyst, possessing desirable catalytic activity and durability simultaneously with a low Pt content, is promising for the future development of low-cost, high-performance alkaline fuel cells. Of course, the strategy to decorate atomic-level Pt species on the defective regions of the metals is not limited to the Co-Pd-Pt system, and can be extended further and make applicable to the other catalyst systems with most types of 3*d* transition metals in the core region.

## Methods

### Catalyst synthesis

Pt_3_ decorated Co-Pd core-shell nanocatalysts were synthesized by a wet chemical reduction method with sequential control. To improve the attachment, CNT support (CNT, Cnano Technology Ltd.) was acid treated in aqueous solution of 4.0 M sulfuric acid at 80 °C for 4 h (corresponding characterization result is presented in Supplementary Fig. 16). The CNT powders were filtered and then washed with distilled water until the pH value of the washing liquid reached 6.0. After washing and drying, the resulting 36 mg of CNT powder was immersed in an aqueous solution of cobalt chloride (CoCl_2_, 99%, MW = 129.84, Sigma-Aldrich Co.) and stirred at 250 rpm at 25 °C for 4 h. This solution (solution A) contained 33.0 mg CoCl_2_ (0.25mmol of Co metal ion) and 6.0 g H_2_O. After immersion, 3.0 g of H_2_O solution containing 20.0 mg (0.54 mmol) NaBH_4_ (99%, Sigma-Aldrich Co.) was added to solution A and stirred at 250 rpm for 10 s. Then, Pd precursor solution comprising 2.0 g distilled water and ~48.4 mg (~0.046 mmol) Pd ion was added in solution A to form solution B. In solution B, the mole ratio of Co/Pd was 1.0. The Pd precursor solution was prepared by dissolving metal powders (Pd, 99%, Sigma-Aldrich Co.) in 1.0 M HCl_(aq)_. After 20 s, 500 mg of aqueous solution containing ~26.6 mg H_2_PtCl_6_•6H_2_O (99%, Sigma-Aldrich Co.) was added to solution B within a reaction time of 10 s (the atomic ratio of Pt/Pd is 1/20). In this step, Pt^4+^ was adsorbed on defect sites of the Pd shell and Co-Pd interface, and then reduced to Pt_3_ by interacting with H.

Synthesis of Co@Pd/CNT nanocatalysts followed similar processes as those used for the preparation Co@Pd-Pt/CNT nanocatalyst, except for the Pt decoration treatment. Co@Pt/CNT and Pd@Pt/CNT nanocatalysts were synthesized by immersing CNT in Co and Pd precursor aqueous solution at 25 °C for 4 h, respectively, followed by the addition of 3.0 g of NaBH_4_, and then 500 mg of Pt precursor solution to the reaction system. More details of sample preparation are provided in Supplementary Information.

### Structure characterization

TEM was performed on a JEOL Grand ARM equipped with two spherical aberration correctors at 300 kV. HAADF-STEM images were recorded using a convergence semi-angle of 22mrad, and inner and outer collection angles of 83 and 165mrad, respectively. XAS, XPS, and UPS experiments were carried out at beamlines BL-17C, 01C1, and BL-24A1 of the National Synchrotron Radiation Research Center (NSRRC, Taiwan). XRD patterns were measured at beamline BL-12B2 of Spring-8, Japan. Model analysis was conducted in the Artemis program with atomic structural models determined in physical structure characterization, and models for data analysis were generated by feff8.0 code.

### Electrochemical testing

As-synthesized nanocatalysts were mixed in a slurry sample and coated on a rotational disk electrode to conduct LSV and CV analyses. The slurry samples were made of 5.0 mg active carbon-supported nanocatalysts mixed in distilled water (14.0 mL), IPA (6.0 mL), and 5 wt% Nafion (0.1 mL). The mixture was stirred at 250 rpm for 30 min prior to ultrasonication at 30 °C for 30 min. To prepare the working electrode in the ORR sweeping experiment, the slurry (20.0μL) was dropped and dried on top of a rotating graphite electrode (with a diameter of 5.0 mm). LSV data were collected using a potentiostat (CH Instruments Model 600B) equipped with a three-electrode electrochemical system. This system consisted of a standard electrode of mercury oxide electrode (the voltage was calibrated by 0.098 V in alignment to that of RHE), a counter electrode of Pt metal wire, and a working electrode. The electrolyte was an aqueous solution of KOH (0.1 M). The voltage scan rate was 5 mV s^−1^ for LSV and 50 mV s^−1^ for the accelerated degradation test. In these tests, the speed of the rotation electrode was 1600rpm.

### First principle calculation

Calculations based on DFT were carried out with the Vienna Ab-initio Simulation Package^42–45^. All the calculations were performed using a plane-wave basis with a cutoff kinetic energy of 420 eV and the projector augmented wave method^46,47^. The exchange-correlation functional was treated at the level of the generalized gradient approximations, using the Perdew–Burke–Ernzerhof functional^48^. The structure of Co@Pd-Pt catalyst was modeled by a five layer of 4 × 4 Co (111) slab capping by one layer of Pd doped with Pt_3_ (Co5L-Pd1L-Pt_3_), which represents the structure of Co@Pd-Pt based on our experimental evidence, particularly, taking the neighboring Co and Pd atoms around Pt_3_ into account. It is worthwhile noting that the (111) slab in face-centered cubic phase is used to keep consistent with the observation from the XRD result (see Supplementary Fig. 9) showing the preferential growth of the (111) facet in the Co@Pd-Pt/CNT catalyst. For comparison, the models of three Pt atoms incorporating into six layers of Pd (111) and three Pt atoms incorporating into six layers of Co(111) were also constructed, respectively. In the calculation part, the Pt atoms were positioned at different sorption sites of the top layer to confirm the most stable atomic configuration. We applied 5 × 5 × 1 Γ-centered *k*-points to map the Brillouin zone and an inter-slab vacuum spacing of 15 Å to avoid the interaction between the replicas of the slabs. The sensitivity of total energies for slab thickness, vacuum spacing, and kinetic energy cutoff were examined as well. All the relevant adsorbates and the upper most four layers of slabs were fully relaxed, while the bottom two layers were fixed to their calculated bulk position.

To investigate the electron localization of Co@Pd-Pt catalyst quantitatively, we introduced the charge density difference distribution (Δ*ρ*), which is defined as Δ*ρ* = *ρ*_T_ − *ρ*_S_ − *ρ*_Pt_. Here, *ρ*_T_ is the charge density of Co_5_LPd_1_L(111)-Pt_3_ model, *ρ*_S_ is the charge density of Co_5_LPd_1_L(111) without Pt_3_ decoration, and *ρ*_Pt_ is the charge density of isolated Pt_3_ at the same position in the model.

The adsorption energy (*E*_ads_) of a single oxygen atom adsorbed on the surfaces of interest is defined as *E*_ads_ = *E*(O − NC) − 1/2*E*(O_2_) − *E*(NC), where *E*(O − NC) is the total energy of the entire system, *E*(O_2_) is the energy of an oxygen molecule, and *E*(NC) is the energy of the pristine slab. The negative values of *E*_ads_ refer to the oxygen adsorption in endothermic.

## Supplementary information

Supplementary Information

## Data Availability

The data that support the findings of this study are available from the corresponding author on reasonable request.
