# Supplementary material for: Platinum-trimer decorated cobalt-palladium core-shell nanocatalyst with promising performance for oxygen reduction reaction
Source: Nat Commun. 2019 Jan 25;10:440. doi: 10.1038/s41467-019-08323-w (PMC6347633; doi:10.1038/s41467-019-08323-w)
Supplement: Supplementary file 1 — Supplementary Information [file 41467_2019_8323_MOESM1_ESM.pdf]

Supplementary Information for

Platinum-trimer decorated cobalt-palladium core-shell nanocatalyst with promising performance for oxygen reduction reaction

Dai et al.

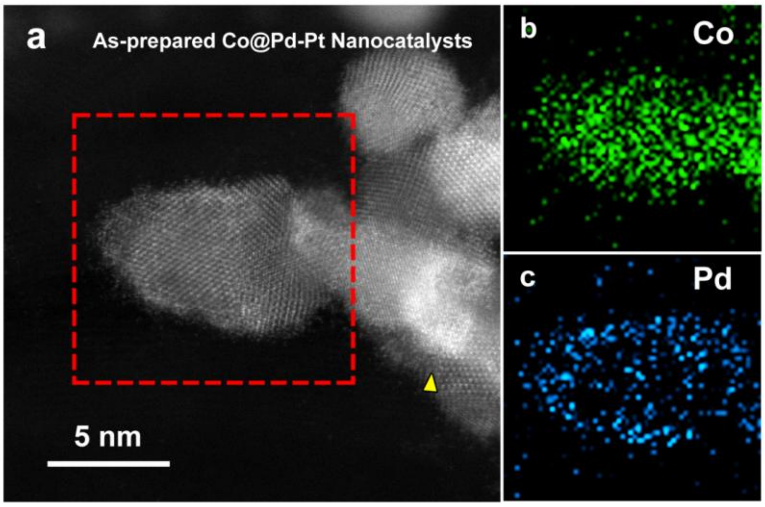

Supplementary Figure 1 | AC-STEM characterization showing the Co core-Pd shell structure of as-prepared Co@Pd-Pt nanocatalysts. a. Atomic-resolution HAADF-STEM image of as prepared nanocatalysts. Yellow arrow indicates the agglomerated Pt species showing highest Z-contrast. Red square region shows a Co core-Pd shell structure. **b, c.** EDS elemental maps showing the distribution of Co and Pd of the nanoparticle in red square region in **a**.

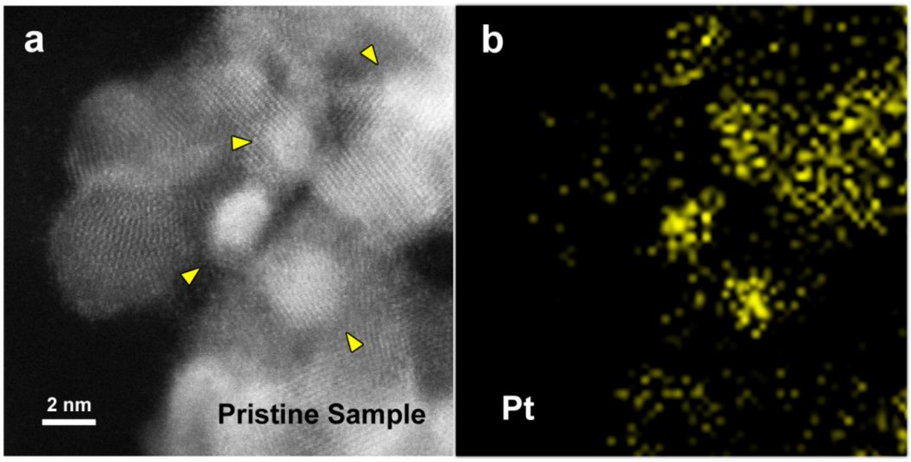

Supplementary Figure 2 | AC-STEM characterization showing the decorated Pt in the as-prepared Co@Pd-Pt/CNT nanocatalysts. a. Atomic-scale HAADF-STEM image. Yellow arrows indicate the decorated Pt species. **b.** EDS elemental maps showing the distribution of Pt.

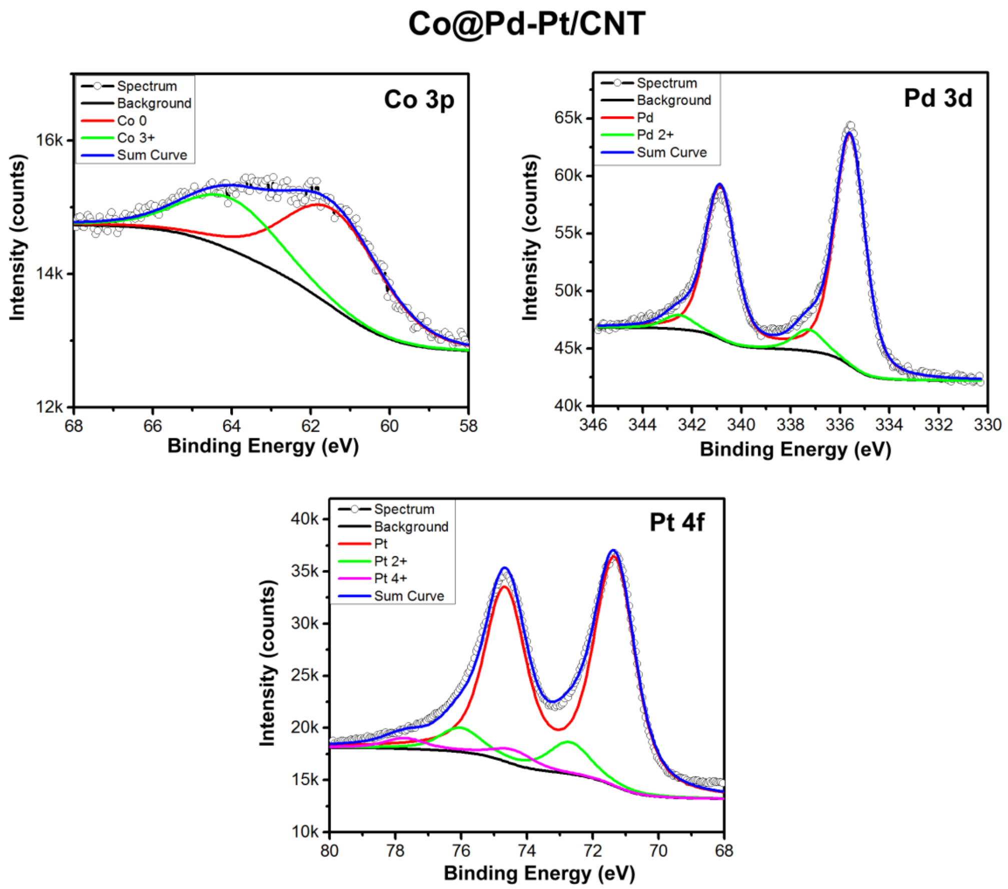

Supplementary Figure 3 | XPS spectra of as-synthesized Co@Pd-Pt/CNT catalysts.

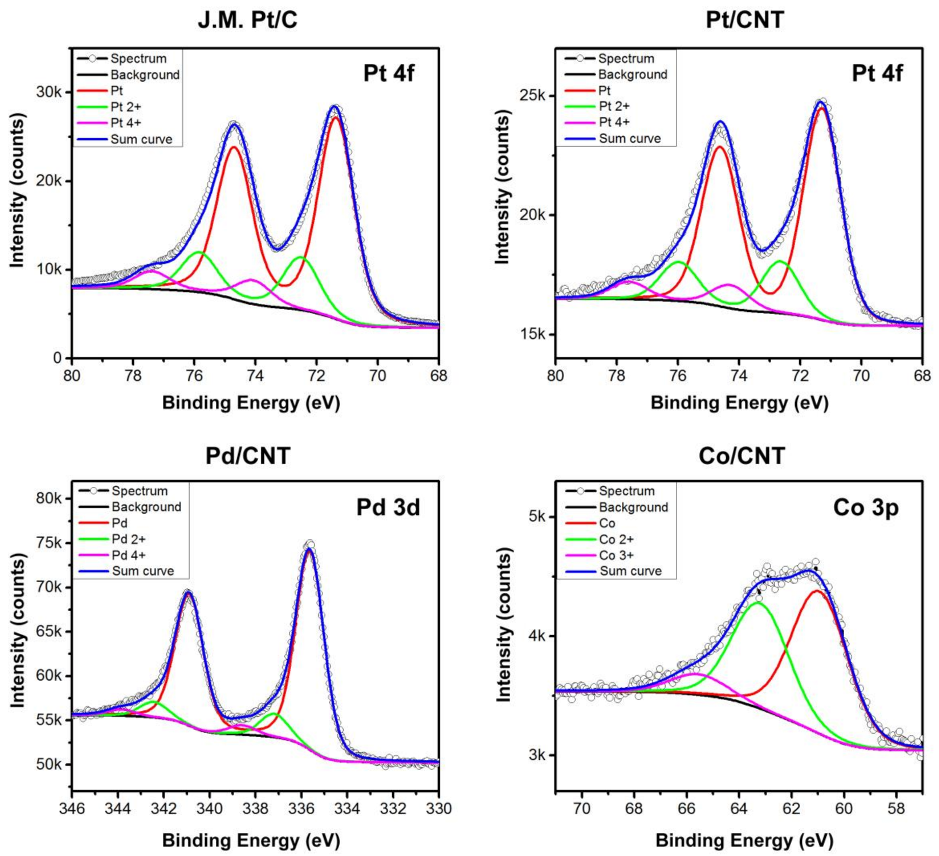

Supplementary Figure 4 | XPS spectra of control samples commercial J.M. Pt/C, Pt/CNT, Pd/CNT, and Co/CNT catalysts.

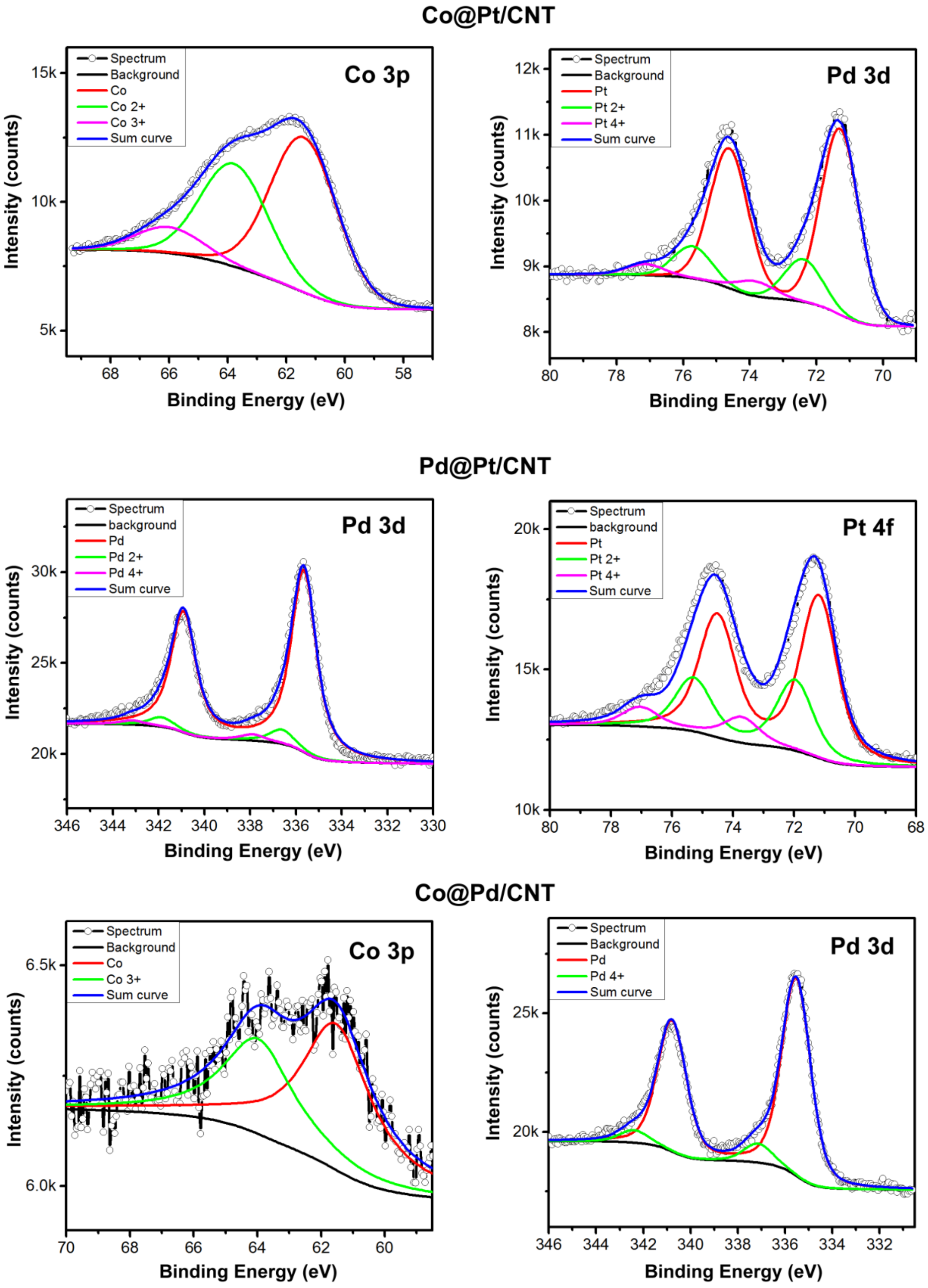

Supplementary Figure 5 | XPS spectra of control samples Co@Pt/CNT, Pd@Pt/CNT, and Co@Pd/CNT catalysts.

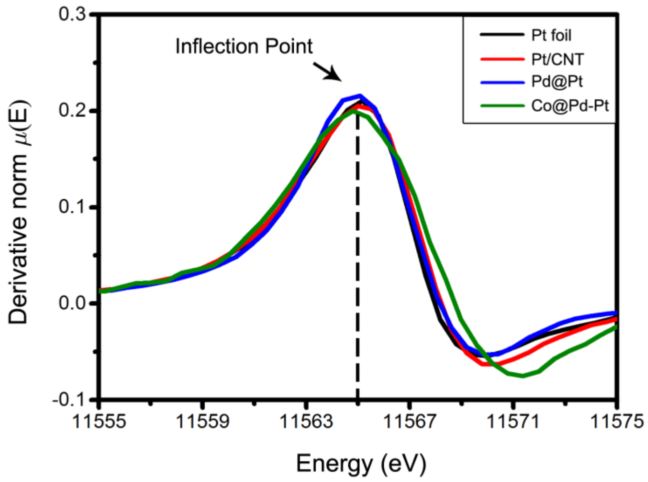

Supplementary Figure 6 | Derivative Pt L_3-edge XANES spectra of Co@Pd-Pt/CNT nanocatalysts compared with control samples and reference sample.

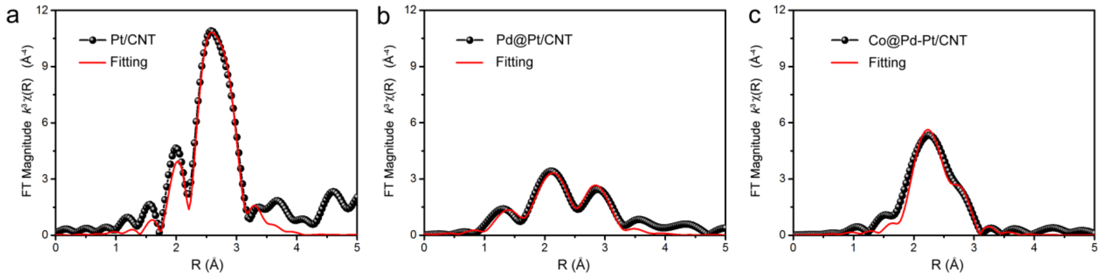

Supplementary Figure 7 | Model analysis of fitting curves compared with experimental FT-EXAFS spectra at Pt L_3-edge of (a) Pt/CNT, (b) Pd@Pt/CNT, (c) Co@Pd-Pt/CNT

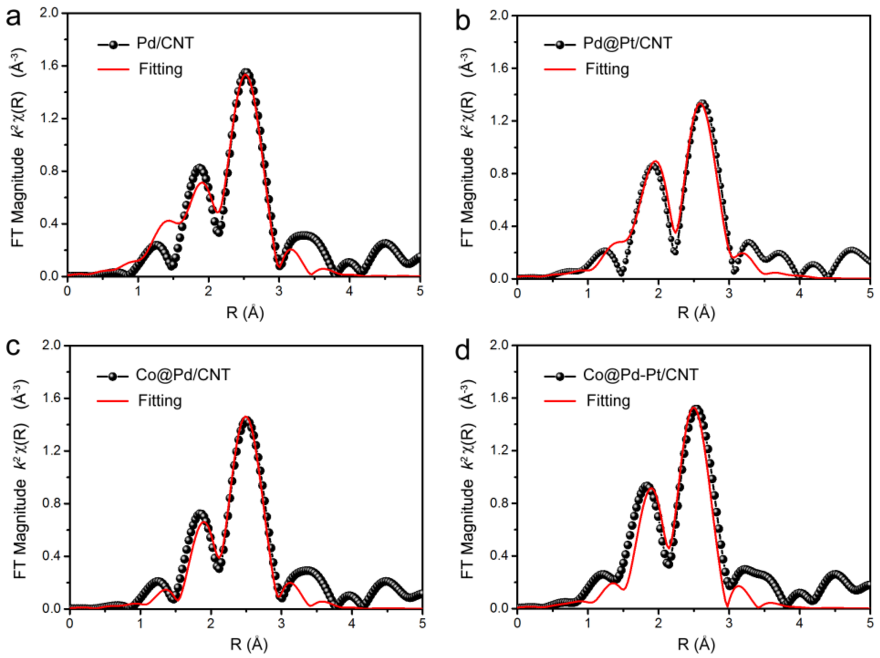

Supplementary Figure 8 | Model analysis fitting curves compared with experimental FT-EXAFS spectra at Pd K-edge of (a) Pd/CNT, (b) Pd@Pt/CNT, (c) Co@Pd/CNT, (d) Co@Pd-Pt/CNT.

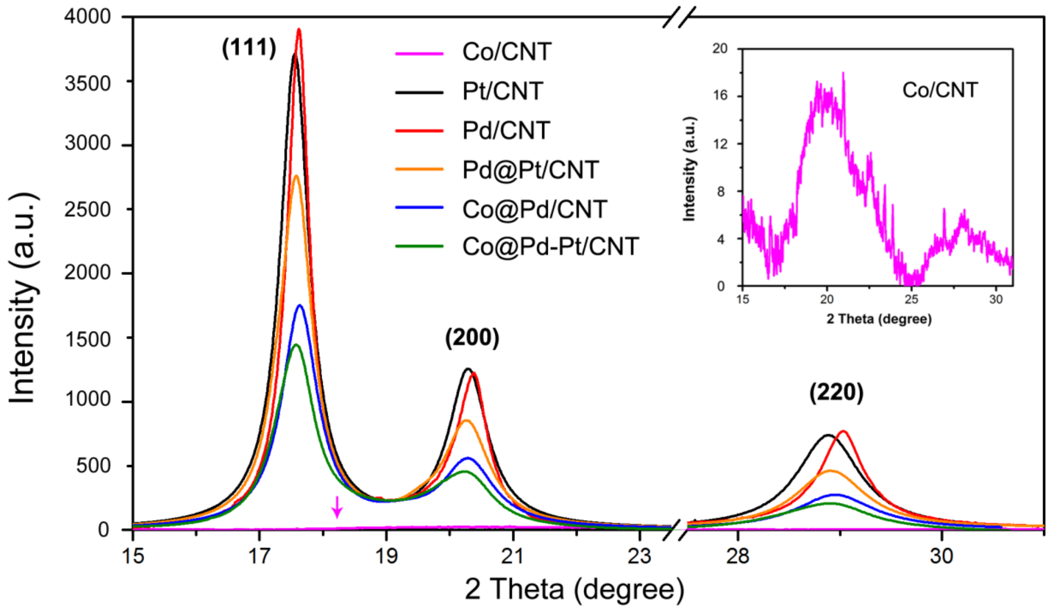

Supplementary Figure 9 | XRD patterns of as-prepared Co@Pd-Pt/CNT nanocatalysts compared with the control samples including Co/CNT, Pd/CNT, Pt/CNT, Co@Pd/CNT, Pd@Pt/CNT catalysts. Co/CNT shows a very weak XRD intensity due to oxidation. Inset is the enlarged region of Co/CNT catalyst with the removal of background of CNT to show the XRD patter of Co part.

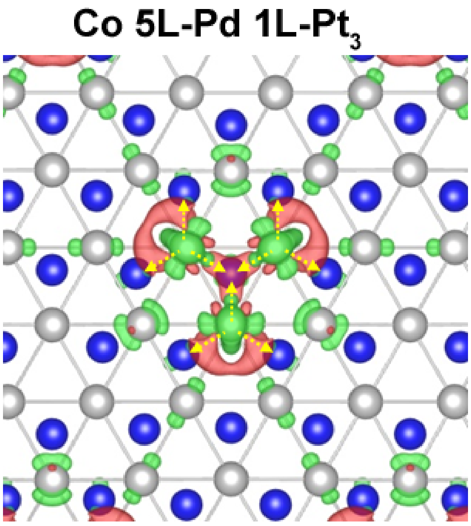

Supplementary Figure 10 | DFT calculation result showing charge density state of Co5L-Pd1L-Pt$_3$ model. The light grey, dark grey (covered by green arches), and blue balls represent Pd, Pt, and Co atoms, respectively. Yellow dashed arrows show that charge localization is around Pt3 in the first layer and neighboring Co atoms in the second layer. The red and green arches represent the distribution of localized charge density and depleted charge density, respectively.

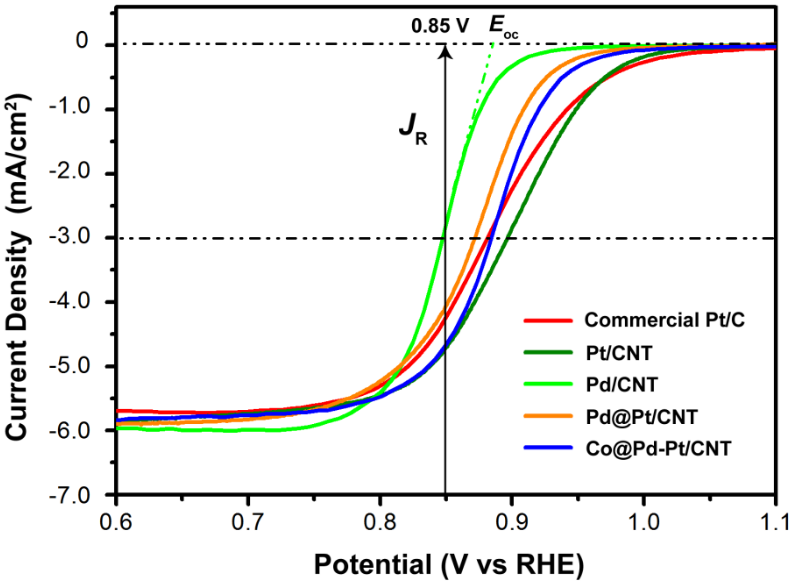

Supplementary Figure 11 | Initial ORR curves of Co@Pd-Pt/CNT nanocatalyst compared with controlled samples.

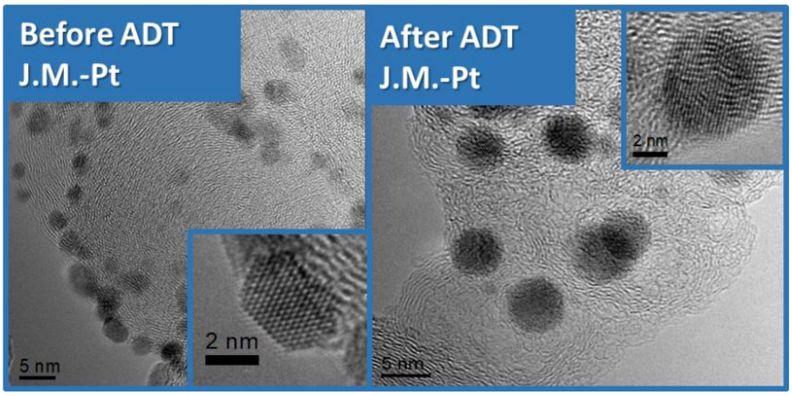

Supplementary Figure 12｜HRTEM results of commercial Pt/C catalyst before and after ADT for 8000 cycles.

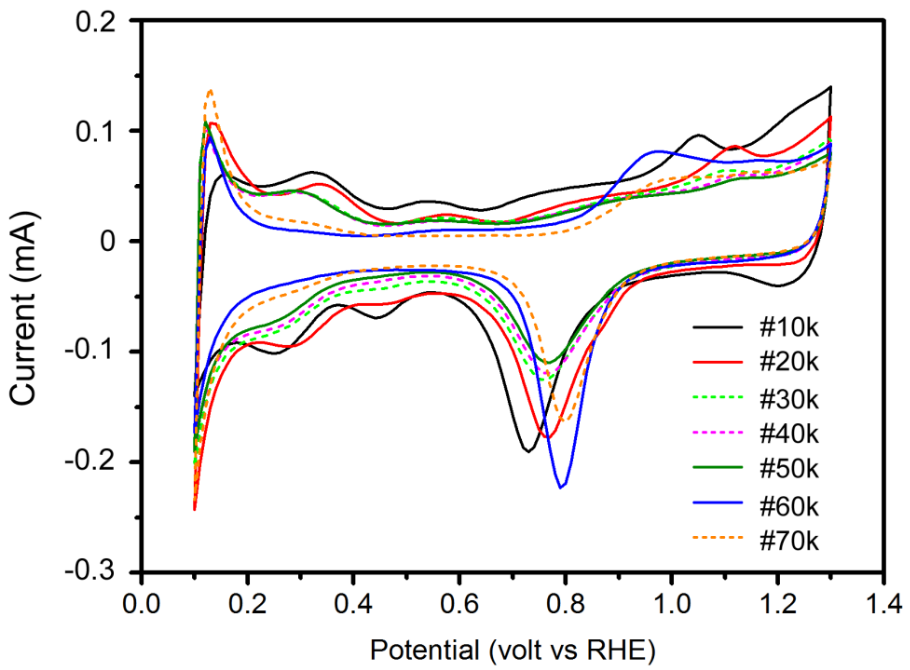

Supplementary Figure 13｜Detailed CV curves of Co@Pd-Pt/CNT nanocatalyst during the first 71k ADT cycles.

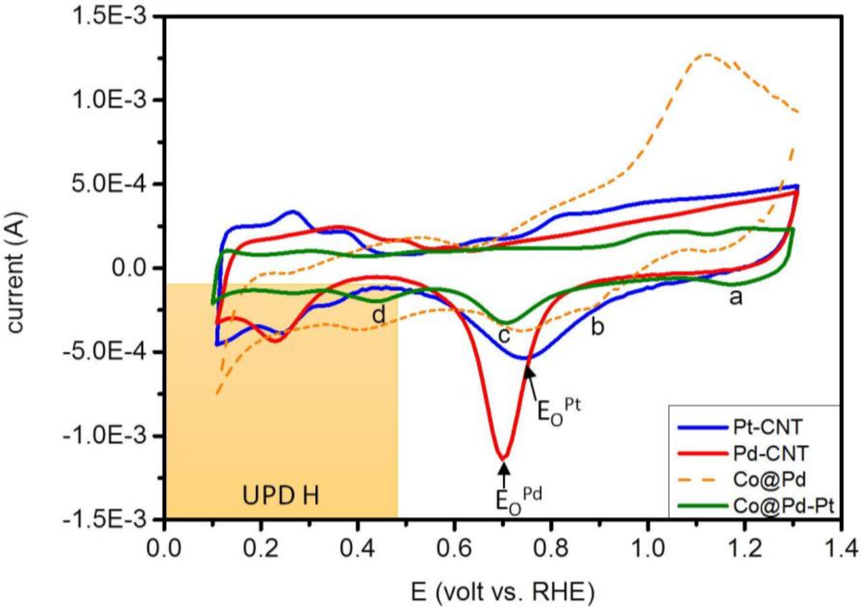

Supplementary Figure 14 | CV sweeping curves of Co@Pd-Pt compared with those of control samples (Pt/CNT, Pd/CNT, and Co@Pd/CNT) at the initial state.

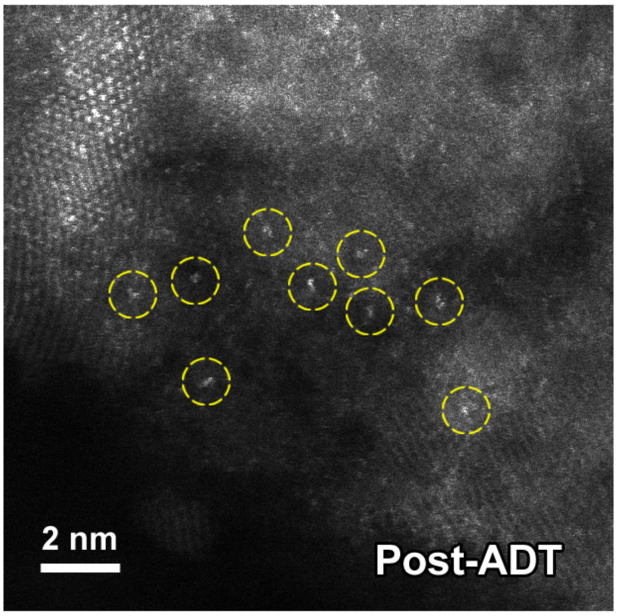

Supplementary Figure 15 | Atomic-resolution HAADF-STEM image of post-ADT Co@Pd-Pt/CNT nanocatalyst. Yellow circles indicate the Pt_3 species.

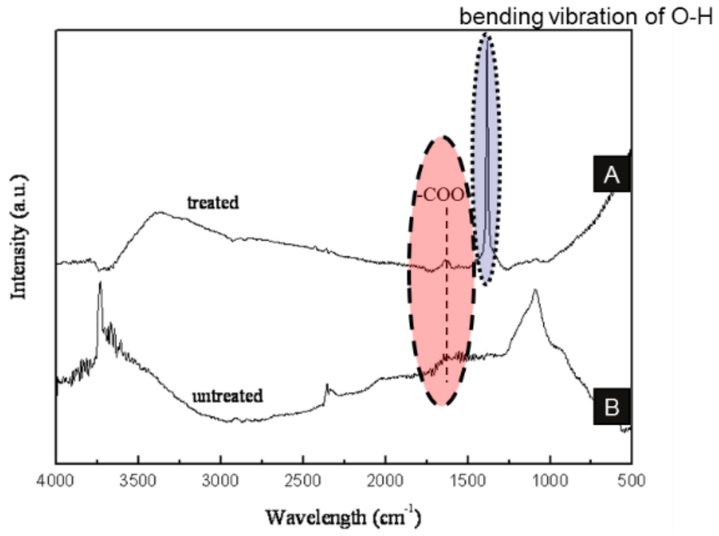

Supplementary Figure 16｜FTIR spectra of the MWCNTs with (A) and without (B) an acid treatment.

Supplementary Note 1 | Details about the CNT supports used for synthesis.

In this study, multi-wall CNTs were purchased from Cnano Technology Ltd. (product number: MWCNT-FT9400). The diameter of these CNTs is about 20–40 nm and the length is 10–20 μm. For a better dispersion of the catalysts, the CNTs were treated in aqueous solution of 4.0M sulphuric acid at 80 oC for 4h. FTIR spectra and HRTEM images of the CNT w/ and w/o acid treatments are compared in Fig. S9 presents the FTIR spectra of the CNTs with and without the acid treatment.

According to our TEM observation, the size of Co@Pd-Pt nanoparticles on the initial CNTs is within a range of 6.0– 8.0 nm. In contrast, the average size of Co@Pd-Pt nanoparticles on treated CNTs is about 4.12 nm. Adsorption of Co2+ ions tend to form at the defective and ligand sites on an acid-treated CNT surface, thus resulting in high densities of Co nuclei for the subsequent heterogeneous crystal growth of Pd crystal. Therefore, the particle size of Co@Pd-Pt nanoparticles on acid-treated CNTs is smaller.

Supplementary Note 2 | Synthesis of Co@Pd-Pt/CNT catalysts

In this study, proper controls of sequences and time in all reaction steps enable the growth of Pt3 decorated Co@Pd core-shell structure. For clarifying the rationales, reaction pathways for all steps are introduced as follow:

Step 1: Chemisorption of Co^{2+} in CNT surface for 4 hours. In this step Co^{2+} ions will be chelated or chemisorbed by ligands in CNT surface. Shown by FTIR analysis (Fig. S9), high contents of ligand (COO^-, C=O, and O-H) might assist chelation and distribution of Co^{2+} in CNT surface.

Step 2: Reduction of Co^{2+}@CNT by interacting with exceed $NaBH_4$ ($NaBH_4$ / Co^{2+} = 4.0 mole ratio) to form solution A. Solution A contains suspension of Co/CoO_x@CNT powder and exceed $NaBH_4$ molecules.

Step 3: After 10 seconds, Pd^{2+} ions are added in solution A. In this step, Pd^{2+} ion in solution and adsorbed in liquid phase will be reduced by $NaBH_4$ into metallic Pd clusters. Pd^{2+} ions adsorbed in Co/CoO_x@CNT surface will be reduced into Pd cluster. Those Pd clusters formed in liquid phase will rapidly adsorb in heterogeneous interfaces (CNT or Co/CoO_x@CNT) or agglomerate into homoatomic crystals.

Considering to kinetics of crystal growth, heterogeneous crystal growth is naturally preferred due to its relatively lower energy barrier as compared to that of homogeneous ones. After reaction, sample of Co@Pd/CNT was placed in room temperature for 10 minutes in order to consume all reduction agents.

Step 4: Formation of Pt clusters on Co@Pd surface by adsorption of Pt^{4+} ions followed by their reduction by $NaBH_4$. With a short adsorption time (10s), galvanic replacement between Pt^{4+} ion and atom in solid phases (Pd, Co, CoO_x) is suppressed. After addition of reduction agent, agglomeration between Pt atoms into nanoclusters of nanoparticles are inhibited due to the strong bonding of Pt atom to adsorbate in which homoatomic clusters between Pt atoms are suppressed due to a presence of high defect density in surface.

Supplementary Note 3 | Calculation of ORR mass activity

The mass activity (MA) is calculated by following equation

$$mass\,activity\,(A\,mg^{-1}) = J_k \times \frac{area}{mass\,of\;catalyst} \quad \text{(eq. 1)}$$

where J_k is the kinetic current density (mA/cm^2) and area is the geometric area of working electrode. The mass activity of the catalyst is estimated via the calculation of J_k and normalization to the catalyst loading (CL) of the disk electrode. In our study, CL is ~0.073 mg (comprising 0.0042 mg of Pt, 0.046 mg of Pd, and 0.0256 mg of Co) and J_k is 12.5 mA cm^{-2} for Co@Pd-Pt/CNT catalyst in the working electrode.

In the manuscript, MA_{Pt} is calculated by dividing total J_k to Pt loading because the MA contribution from Co@Pd structure is very limited, according to the electrochemical results of the control samples Co/CNT and Co@Pd/CNT catalysts (Table S5). It can be found that the Co/CNT catalyst exhibit no ORR activity at 0.85 V vs. RHE. Moreover, J_k of the control sample Co@Pd/CNT is 5.15 mA/cm^2, and the corresponding MA contribution from Pd is very limited, only 72.2 mA/mg$_{Pd}$. Thus, considering the similar Pd content in the Co@Pd/CNT and Co@Pd-Pt/CNT samples,

it is reasonable to conclude that main ORR activity of the Co@Pd-Pt/CNT catalyst definitely comes from the decorated Pt_3 species.

Supplementary Note 4 | CV analysis of Co@Pd-Pt and control samples.

The discussion is based on the comparison of CV curves of Co@Pd-Pt/CNT and various control samples (Pt/CNT, Pd/CNT, Co@Pt/CNT) and DFT calculation outputs. Here, Table S5 summarizes the detailed DFT results of the adsorption energy of O atoms on different adsorption sites of various catalyst surfaces.

Accordingly, E_{ads}^{O} is -0.82 eV for Pt_3 site in a Pt (111) facet. For oxygen adsorption in a Pd (111) facet, E_{ads}^{O} is -1.15 eV for Pd3 site. As compared to that of Pt_3 in Pt (111) facet, E_{ads}^{O} of Pd_3 in Pd (111) facet is increased by -0.33 eV suggesting the higher oxidation extent of Pd metal. For Co (111) facet, E_{ads}^{O} is 1.8 eV. Such a value is similar to that of ionic bond or covalent bond in a metal oxide. It indicates a preference of oxide formation in Co nanoparticle and is consistently explained by the absence of reaction peaks in backward sweep of Co/CNT. From design of crystal growth scenario in this study, co-existence of Co@Pt and Co@Pd is possible in Co@Pd-Pt sample and, therefore, E_{ads}^{O} in Pt_3 site of Pt decorated Co (Co6L-Pt3), Pt_3 site of Pt decorated Pd (Pd6L-Pt3), and Pd_3 site of Pd decorated Co (Co6L-Pd3) is calculated. As compared to that of Pt3 in Pt(111), E_{ads}^{O} of Pt3in Co6L-Pt3 is decreased by 0.095 eV (-0.725 eV) indicating a weakening of Pt-O bond in Co6L-Pt3. Such a weakening could be attributed to a strong attraction from neighboring sites (including Pt2Co, PtCo2, Co3) of Pt3 in a Co6L-Pt3 model. Again, due to the strong attraction force of Co to O atom, Co6L-Pd3 possesses a similar E_{ads}^{O} tend to that of surface sites in Co6L-Pt3. For Pd6L-Pt3, E_{ads}^{O} is -1.004 eV in Pt3. Compared to E_{ads}^{O} of Pt3, E_{ads}^{O} of Pt2Pd, PtPd2, and Pd3 sites is determined to be -1.265, -1.119, and -1.352 eV, respectively. For the cases of Pd6L-Pt3 and Co5LPd1L-Pt3, trend of E_{ads}^{O} distribution is the same but is lower to that of Co6L-Pt3 surface. Given that oxygen inertia of Pd is higher than that of Co, the stronger E_{ads}^{O} of Pt3 can be explained by the weaker attraction force from its neighboring sites both in Co5LPd1L-Pt3 and Pd6L-Pt3 as compared to that of Co6L-Pt3.

In the backward sweeping in KOH electrolyte, position of reaction peaks refers

to voltage to be applied to desorb O from the catalyst surface. As a result, reaction peaks at a potential higher than E_O^{Pt} refer to a current response of oxygen desorption from adsorption site weaker than Pt-O. Based on such a scenario and results of DFT calculation, as shown in Fig. S10, reaction peak at the highest voltage (a) might be attributed to the oxygen desorption from Pt_3 sites on Co surface in Co@Pd-Pt. The subsequent broad peaks at (c) 0.708 and (d) 0.441 volts (vs. RHE) are sequentially assigned as O desorption reaction from Pd site and $PdCo_{rich}$ site in Co@Pd-Pt, respectively. It is notable that, E_{ads}^{O} at Co site is higher than that of experimental potential range, therefore, O desorption peak doesn't appear in CV curves. The detailed assignment is summarized in Table S6. Notably, deviations between experimental applied potential difference (ΔE_{des}) and DFT calculated E_{ads} could be introduced from the effects of defects and roughness to O adsorption energy in Co@Pd-Pt surface.

Supplementary Note 5 | Discussion of the size of Pt species in the post-ADT Co@Pd-Pt/CNT catalyst.

The averaged diameter of the single Pt atoms in the post-ADT Co@Pd-Pt catalyst is about 173 pm, which is consistent with the published data (176 pm) in the literature (*JACS* 2017, 139, 14150). In contrast, the measured diameter of the Pt_3 species is within the range of 314~386 pm, showing an obvious difference from the size of single Pt atoms.

Supplementary Table 1 | XPS fitting results of as-synthesized catalysts (probing depth is around 1.5 nm).

Sample	Pt chemical states			Pd chemical states			Co chemical states			Composition (%)		
	Pt 0	Pt 2+	Pt 4+	Pd 0	Pd 2+	Pd 4+	Co 0	Co 2+	Co 3+	Pt	Pd	Co
**J.M.-Pt/C**	72.4	19	8.6	-	-	-				100	-	
**Pt/CNT**	73.7	18.2	8.1	-	-	-				100	-	
**Pd/CNT**	-	-	-	85.8	10.1	4.1				-	100	
**Co/CNT**							53	7.7	39.3			100
**Co@Pt/CNT**	82.5	13	4.5	-	-	-	51.6	9.2	39.2	36.3		63.7
**Pd@Pt/CNT**	75.4	16.7	7.9	87.7	8.3	4				34.4	65.6	
**Co@Pd/CNT**	-	-	-	93.4	6.6	0	60.3	0	39.7		97.1	2.9
**Co@Pd-Pt/CNT**	83.3	11.6	5.1	91.5	8.5	0	65.4	0	34.6	46.9	49.2	3.9

**Supplementary Table 2 | ** Average size of various catalysts by using AC-STEM.

**Sample**	Pt/CNT	Pd/CNT	Co@Pd/CNT	Pd@Pt/CNT	Co@Pd-P/CNT
**Diameter (nm)**	4.91	5.93	4.65	4.30	4.12

**Supplementary Table 3 | ** Structure parameters of the catalyst samples determined by XRD.

Sample	index	2θ	$H_{(111)}/H_{(hkl)}$
Pt/CNT	(111)	17.56	
	(200)	20.26	2.85
	(220)	28.87	4.78
Pd/CNT	(111)	17.61	
	(200)	20.33	3.31
	(220)	29.01	5.27
Pd@Pt	(111)	17.54	
	(200)	20.21	3.29
	(220)	28.90	5.91
Co@Pd	(111)	17.62	
	(200)	20.24	3.24
	(220)	28.96	6.65
Co@Pd-Pt	(111)	17.57	
	(200)	20.28	3.22
	(220)	28.86	6.73

Supplementary Table 4 | Electrochemical performances of commercial Pt/C (J.M.-Pt/C), Pt/CNT, and Co@Pd-Pt/CNT catalysts.

Sample	E_{oc} (V vs. RHE)	N	ECSA_O (m^2 g^{-1})	MA_{Pt} (mA /mg $_{Pt}$)	ADT cycles
J.M.-Pt/C	0.956	4	20.7	67.1	31k
Pt/CNT	0.935	4.1	21.9	271.9	12k
Co@Pd-Pt/CNT	0.944	3.8	32.5	2055.1	310k

* N: charge transfer number calculated at 0.5 V vs. RHE

*The final ADT is defined at the cycle when the MA degraded more than 25% of one catalyst (except for Co@Pd-Pt/CNT).

Supplementary Table 5 | Calculated mass activity of various control samples and Co@Pd-Pt/CNT catalyst.

Sample	J_k (mA/cm^2)	MA_{Pt} (mA/mg$_{Pt}$)
J.M.-Pt/C	5.34	67.1
Pt/CNT	15.22	271.9
Pd/CNT	1.49	32.7*
Co/CNT	0	0
Co@Pd/CNT	5.15	72.2*
Co@Pd-Pt/CNT	12.5	2055.1

* MA of Pd/CNT and Co@Pd/CNT are calculated with the Pd weight.

* MA of all samples is calculated at 0.85 volt (vs. RHE).

Supplementary Table 6 | Comparison of ORR performance between Co@Pd-Pt catalyst and other Pt-decorated catalysts (in 0.1 M KOH media) from literature.

**Catalysts**	**E_{onset} vs RHE**	**E_{half} vs RHE**	**ADT cycles**	**Durability**	**References**	**MA_{Pt} (mA/mg_{Pt})**
Pt_3 decorated Co@Pd /CNT	0.944	0.88	310k	No penalty	This study	2055 @ 0.85V
H-Pt cluster decorated $CaMnO_3$ nanoparticles	0.95	0.81	6k	95% retention of current density at 0.80 V for 27.8 h	*Adv. Mater.* 26, 2047-2051 (2014)	380 @ 0.85V
Pt@Pd nanocubes	0.97	0.88	1k	80 % retention of current density	*J. Power Sources* 268, 712−717 (2014)	N/A
Co@Pt nanoparticles encapsulated in nitrogen-doped porous carbons	N/A	N/A	5k	17 mV penalty	*J. Power Sources* 343, 458-466 (2017)	71.9 @ 0.85 V
Pt-decorated three dimensional N-doped carbon	N/A	N/A	3k	81% retention of current density	*Carbon* 128, 38-45 (2018)	162.88@0.9V
Pd@PtNi/MWCNT	N/A	N/A	5k	No penalty	*J. Power Sources* 365, 26-33 (2017)	73.3

Supplementary Table 7 | DFT calculation determined adsorption energy of O atoms at adsorption sites of different models.

**Model**	**site**	E_{ads}^{O} **(eV)***	$\Delta E_{ads} = E_{ads}^{O}(Pt) - E_{ads}^{O}(peak)$
**Pt(111)**	Pt_3	-0.82	
**Pd(111)**	Pd_3	-1.15	0.33
**Co(111)**	Co_3	-2.62	1.8
$\mathbf{Co_{6L}\text{-}Pt_3}$	Pt_3	-0.725	-0.095
	Pt_2Co	-1.272	0.452
	$PtCo_2$	-1.843	1.023
	Co_3	-2.393	1.573
$\mathbf{Co_{6L}\text{-}Pd_3}$	Pd_3	-0.984	0.164
	Pd_2Co	-1.59	0.77
	$PdCo_2$	-2.425	1.605
	Co_3	-2.441	1.621
$\mathbf{Pd_{6L}\text{-}Pt_7}$	Pt_3	-1.003	0.183
	Pt_2Pd	-1.265	0.445
	$PtPd_2$	-1.119	0.299
	Pd_3	-1.352	0.532
$\mathbf{Co_{5L}Pd_{1L}\text{-}Pt_3}$	Pt_3	-0.65	-0.17
	Pt_2Pd	-0.64	-0.18
	$PtPd_2$	-0.97	0.15
	Pd_3	-0.87	0.05

* E_{ads}^{O} of hcp-type adsorption site; E_{ads}^{O} (Pt): E_{ads}^{O} of Pt_3 site in Pt (111)

Supplementary Table 8 | Oxygen desorption peak position of various catalysts.

Catalyst	O desorption peak	(V vs. RHE)	$\Delta E_{des} = E_O^{Pt} - E_{peak}$	Site
**Pt/CNT**	E_O^{Pt}	0.745	0	Pt
**Pd/CNT**	E_O^{Pd}	0.702	0.043	Pd
**Co@Pd**	a	1.134	-0.389	Pd_{rich}-Co
	b	0.888	-0.143	Pd-Co
	c	0.734	0.011	Pd
	d	0.409	0.336	Pd-Co_{rich}
**Co@Pd-Pt**	a	1.173	-0.428	Pt-Co_{rich}
	c	0.708	0.037	Pd
	d	0.441	0.304	Pd-Co_{rich}

**Supplementary Table 9 | ** DFT calculated adsorption energy of chemisorbed O atom (O^{ads}) on models. The top, bridge, hcp-hollow, and fcc-hollow sites are denoted as *top*, *bri*, *hcp*, and *fcc*, respectively. The sites between top and fcc are denoted as *tf*, and the sites between top and hcp are denoted as *th*.

O^{ads}		
**model**	site	E_{ads}(eV)
**$Co_{5L}Pd_{1L}$-Pt_3 (111)**	Pd_*top*	0.32
	Pd_*tf*	-0.12
	Pd_*th*	-0.39
	Pt_*th*	-0.03
	Pt_*top*	-0.12
	Pt_*tf*	-0.49
	PdPd_*bri*	-0.41
	PtPd_*bri*	-0.54
	PtPt_*bri*	-0.54
	Pt3_*hcp*	-0.64
	PtPd2_*hcp*	-0.79
	Pd3_*hcp*	-0.85
	Pt2Pd_*fcc*	-0.63
	Pd3_*fcc*	-0.84
	PtPd2_*fcc*	-0.95
**Pt (111)**	*hcp*	-0.82
	fcc	-1.24
**Pd (111)**	*hcp*	-1.15
	fcc	-1.34
**Co (111)**	*hcp*	-2.62
	fcc	-2.55

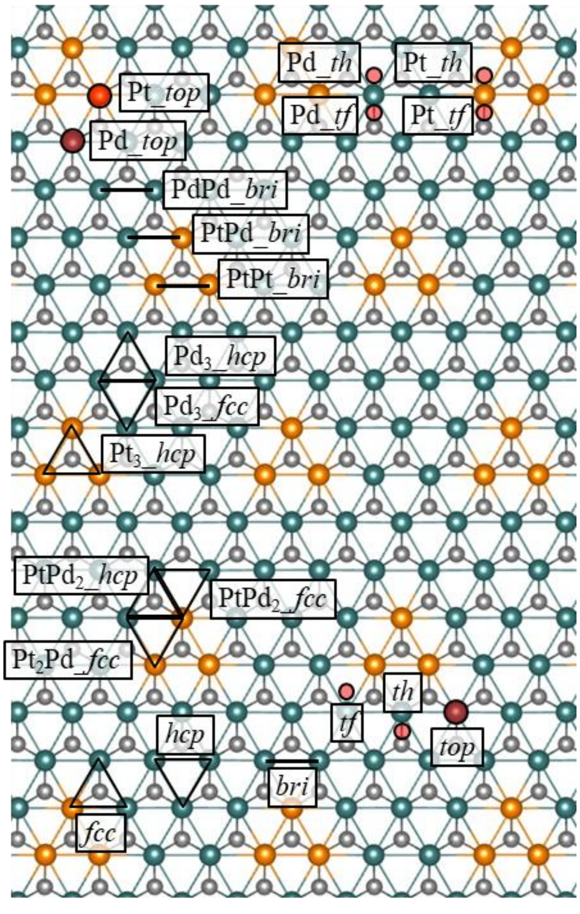

**Supplementary Table 10 | ** DFT calculated adsorption energy of chemisorbed O_2 (O_2^{ads}) molecule on models. The top, bridge, hcp-hollow, and fcc-hollow sites are denoted as *top*, *bri*, *hcp*, and *fcc*, respectively. The sites between top and fcc are denoted as *tf*, and the sites between top and hcp are denoted as *th*.

O_2^{ads}			
**model**	O_2 orientation	site	E_{ads}^{O2} (eV)
**$Co_{5L}Pd_{1L}$-Pt_3(111)**	⊥ to the surface	Pt3_*hcp*	-0.20
		PtPt_*bri*	-0.18
		Pd3_*fcc*	-0.12
		PdPd_*bri*	-0.06
		Pd3_*hcp*	-0.12
		Pt2Pd_*fcc*	-0.22
		PtPd2_*hcp*	-0.12
		Pd_*top*	-0.67
	∥ to the surface	O_2∥PtPt_*bri*	-0.57
		O_2∥PdPd_*bri*	-0.60
		O_2⊥PtPt_*bri*	-0.17
		O_2⊥PdPd_*bri*	-0.73
**Pt(111)**	⊥ to the surface	*hcp*	0.33
		fcc-V	0.23
		fcc-H	-0.69
	∥ to the surface	O_2 ∥ *bri*	-0.67
		O_2 ⊥ *bri*	O_2 split
**Pd(111)**	⊥ to the surface	*hcp*	-0.15
	∥ to the surface	*bri*	-0.77

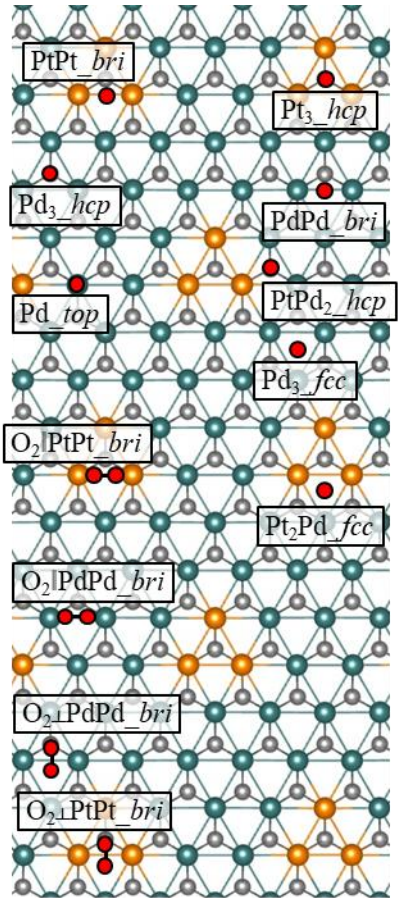

Pt; Pd, 1st layer; Co, 2nd layer; O_2 ⊥ to the surface; O_2 ∥ to the surface
